# Isolation and Evaluation of *Rhizopus arrhizus* Strains from Traditional Rice Wine Starters (*Jiuqu*): Enzyme Activities, Antioxidant Capacity, and Flavour Compounds

**DOI:** 10.3390/foods14020312

**Published:** 2025-01-17

**Authors:** Bo Wan, Tian Tian, Ying Xiong, Siqi Wang, Xinyu Luo, Weifang Liao, Pulin Liu, Lihong Miao, Ruijie Gao

**Affiliations:** School of Life Science and Technology, Wuhan Polytechnic University, Wuhan 430023, China; bo.wan@foxmail.com (B.W.); t15137029987@163.com (T.T.); 17373604579@163.com (Y.X.); m17382200228@163.com (S.W.); 13339704218@163.com (X.L.); leesalwf89@126.com (W.L.); plliu@whpu.edu.cn (P.L.)

**Keywords:** *Rhizopus arrhizus*, Chinese autochthonous strains, isolation and screening, oenological properties, antioxidant capacity, flavour compounds, rice wine

## Abstract

Seventy-eight autochthonous strains of *Rhizopus arrhizus* were isolated from rice wine starter samples across twenty-nine regions in China to evaluate their potential in traditional rice wine fermentation. Strains were assessed for enzyme activity, antioxidant properties, amino acid production, and volatile flavour compounds. Significant variation in enzyme activities was observed, with acidic protease activity ranging from 280 to 1023 U/g, amylase from 557 to 1681 U/g, and esterase from 370 to 2949 U/g. Strains W17 and W42 exhibited the highest enzyme activities and antioxidant capacities, with a total phenolic content of 828 mg/L, total flavonoids of 215 μg/L, and an ABTS scavenging rate of 96.3%. They also produced high levels of glutamic acid (up to 3083 mg/L), enhancing the flavour profile. Histamine levels were low, ranging from 8 to 205 μg/L, ensuring product safety. Analysis of volatile compounds identified 80 substances, including 16 key aroma-active compounds, contributing to a complex flavour profile. These results provide a basis for selecting *R. arrhizus* strains to optimise rice wine fermentation, addressing market demand for diverse and functional products.

## 1. Introduction

Rice wine, one of the oldest traditional fermented beverages, has a history spanning thousands of years. With its distinctive flavour and high nutritional value, rice wine plays a significant role in both the diet and socio-cultural life of many communities, making it a key component of cultural heritage. It is especially popular in East Asia [1,2,3,4,5]. Rich in amino acids [6], vitamins [7], and polyphenols [8], rice wine not only boasts a complex flavour profile but also provides various health benefits, such as antioxidant, anti-inflammatory, and cardioprotective effects [8,9,10]. Notably, its antioxidant properties are of particular importance, as oxidative stress is closely linked to several pathological conditions, including cardiovascular disease [11], ageing [12], and cancer [13], positioning rice wine as a potentially health-promoting beverage [14,15,16].

The fermentation of rice wine involves two primary steps: saccharification and fermentation. During these stages, microorganisms and enzymes in the fermentation starter (*Jiuqu*) contribute to the production of metabolites, imparting unique aromatic and sensory characteristics to the wine [17]. The microorganisms involved include bacteria, moulds, and yeasts, with the mould *R. arrhizus* (= *R. oryzae* [18]) serving as a core strain [19,20,21,22]. However, the traditional use of mixed microbial starters in Chinese fermented alcohol production presents challenges in control and reproducibility. Extended production cycles and complex microbial compositions can lead to the generation of harmful substances such as fusel alcohols and mycotoxins [23]. Additionally, environmental factors significantly influence the microbial community in traditional starters, resulting in variations in flavour and making it difficult to ensure product safety [24]. By selecting and optimising specific microbial strains, the fermentation process can be better controlled, enhancing both product quality and safety, and aligning with the demands of modern industrial production [25].

*R. arrhizus* is recognised as a key industrial fermentation microorganism and has been granted a Generally Recognized as Safe (GRAS)status by the U.S. Food and Drug Administration (FDA). It is characterised by sparse rhizoid structures and regularly shaped sporangia [26,27]. Extensive studies on its morphology, molecular phylogenetics, and biochemistry highlight its genetic complexity and morphological diversity [26,28,29,30,31,32,33,34]. *R. arrhizus* is widely distributed and commonly used in the fermentation of alcohol, grains, fruits, and legumes [35,36,37,38,39]. Its strong substrate absorption capabilities make it an ideal candidate for fermenting agricultural and industrial by-products [40,41]. Its adaptability to diverse environmental conditions has made it indispensable in numerous industrial processes, particularly in the utilisation of raw materials.

In China, *R. arrhizus* is extensively used in the production of traditional alcoholic beverages, such as Maotai, black glutinous rice wine, sweet rice wine, and barley wine [42,43,44,45]. This application is primarily due to its ability to produce amylase and saccharifying enzymes, which break down starch into glucose [45], and generate flavour-enhancing substances such as lactic acid [46] and higher alcohols [47]. These metabolites contribute to the distinctive aroma and flavour of traditional Chinese alcoholic beverages. In recent years, pure fermentation technology has gained traction as it allows better control of the fermentation process by selectively utilising specific microorganisms, ensuring product quality and safety [48,49,50]. This technology ensures a pure fermentation process by preventing unwanted microbial contamination, reducing harmful substances such as fusel alcohols and mycotoxins, and enhancing food safety. Moreover, it supports the automation and standardisation of production, improving industrial efficiency and ensuring consistency. Thus, selecting high-quality *R. arrhizus* strains for pure fermentation is essential to improving rice wine quality and ensuring safety [51]. Through strain selection, strains with high enzyme activity, stable metabolic traits, and environmental adaptability can be obtained, providing reliable microbial resources for industrial production.

This study aims to screen *R. arrhizus* strains suitable for rice wine fermentation from traditional *Jiuqu* and systematically evaluate their fermentation performance to optimise the rice wine production process and enhance both product flavour and stability. Specifically, the study will screen *R. arrhizus* strains from various sources and compare their physicochemical properties, functional active substances, and production of organic acids and flavour compounds in fermented alcoholic beverages. By conducting a comprehensive evaluation, this study seeks to identify strains most suited for industrial rice wine production and provide a scientific basis for future strain improvement and fermentation process optimisation. These findings will support the enhancement of rice wine production efficiency and product quality, further promoting the modernisation and standardisation of the rice wine industry.

## 2. Materials and Methods

### 2.1. Experimental Materials

The raw material, glutinous rice, was sourced from a local farmers’ market in Zhuhu, Xiaogan, Hubei. Reagents for gas chromatography (GC), Gas Chromatography–Mass Spectrometry (GC-MS), and High-Performance Liquid Chromatography (HPLC) were purchased from Sigma-Aldrich, while general analytical reagents were supplied by Shanghai Aladdin Biochemical Technology Co., Ltd., Shanghai, China. The culture media, comprising Malt Extract Agar (MEA) and potato dextrose agar (PDA), were supplied by Qingdao Hi-Tech Industrial Park Hope Bio-Technology Co., Ltd., Qingdao, Shandong, China.

### 2.2. Sample Collection

**Figure 1:**
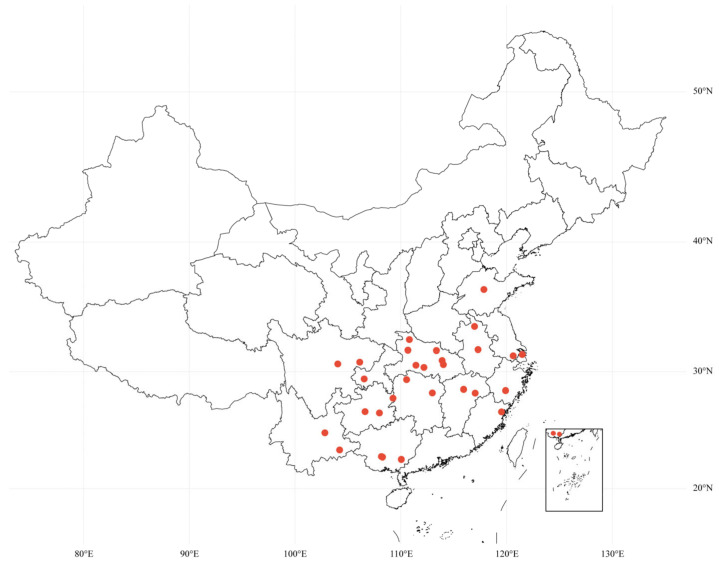
Sampling locations for *R. arrhizus* strains isolated from multiple regions across China. The map was generated using the online platform https://www.bioinformatics.com.cn (Version 1.0, accessed on 3 June 2024).

A total of 78 *Jiuqu* samples, designated W1 through W78, were collected from 29 regions across China (Figure 1), representing the diverse geographic and cultural origins of traditional rice wine fermentation practises. The strains isolated from these *Jiuqu* samples are autochthonous, shaped by long-standing local fermentation traditions. All *Jiuqu* samples were stored at 4 °C for subsequent analysis.

### 2.3. Isolation of Rhizopus

Samples (10 g) were placed in sterile bottles with 90 mL of sterile saline and shaken at 160 rpm at room temperature for 30 min to elute microorganisms. Subsequently, 0.1 mL of serially diluted samples were plated on Martin-Bengal agar with 100 mg/L chloramphenicol to inhibit bacterial growth. After incubation at 30 °C for 3 days, colonies were selected, purified, and preserved on potato dextrose agar (PDA) slants at 4 °C for further use.

### 2.4. Morphological, Sporulation Ability, and Molecular Identification of Rhizopus

Isolated strains were cultured on PDA medium at 28 °C for 5 days to observe colony morphology. Colonies with a creamy, slightly greenish appearance, convex shape, and opaque texture, along with white or greenish reverse sides, were examined under a light microscope. Following the criteria of Zheng et al. [26], they were preliminarily identified as *R. arrhizus*.

Subcultures from 78 strains were grown on 5% MEA at 30 °C for three days, with strain W17 selected as a representative for detailed morphological analysis. Light microscopy observations were performed using a Nikon Eclipse 80i microscope and lactic acid phenol cotton blue (Qingdao Hi-Tech Industrial Park Hope Bio-Technology Co., Ltd., Qingdao, Shandong, China) as the mounting medium, and images were captured with a Nikon DS-Ri1 digital camera. Morphological characteristics were further examined using a scanning electron microscope (SEM; Model SU8100, Hitachi High-Technologies Corporation, Tokyo, Japan).

Sporulation was evaluated on 5% MEA medium using mycelial portions and fresh spores from three-day-old cultures incubated at 30 °C. Observations were made on days two, three, and four, with sporulation levels (A to C) defined macroscopically by culture plate colour and documented using an Apple iPhone 13, based on the method of Dolatabadi et al. [52].

Molecular identification was conducted by sequencing the internal transcribed spacer (ITS) region. Genomic DNA was extracted using the Genomic DNA Purification Kit D5542 (Omega Bio-Tek, Inc., Norcross, GA, USA) following the manufacturer’s instructions. The ITS region was amplified with primers ITS1 and ITS4 as described in [53]. PCR products were confirmed on a 1% agarose gel and sequenced by Sangon Biotech (Shanghai) Co., Ltd., Shanghai, China. Homology of the ITS region was analysed using BLAST (Version 2.10.0) against GenBank.

Phylogenetic trees were constructed using IQ-TREE2 for maximum likelihood analysis of the ITS region [54], with the optimal nucleotide substitution model selected by IQ-TREE’s model test (-m TEST) based on Bayesian Information Criterion scores. Tree visualisation and annotation were performed in FigTree (Version 1.4.4, accessed on 2 September 2024, http://tree.bio.ed.ac.uk/software/figtree), with final adjustments in Inkscape (Version 1.4.0).

### 2.5. Evaluation of Enzyme Production Ability of R. arrhizus

**Figure 2:**
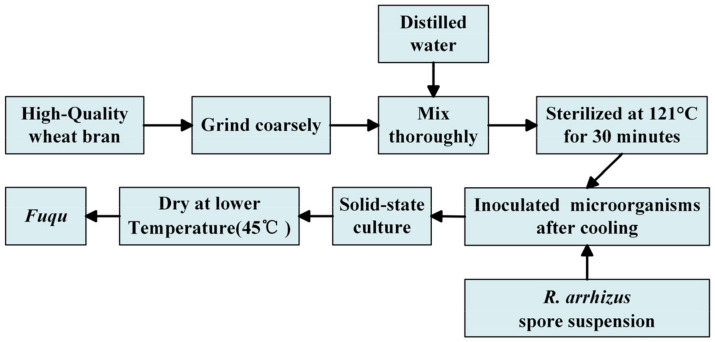
Production process of *Fuqu*.

The *Fuqu* production process (Figure 2) followed the method of Yu et al. [55]. High-quality bran was mixed with water (1:1 *w*/*v*) and steamed at 115 °C for 30 min. After cooling to 30 °C, the bran was inoculated with an *R. arrhizus* spore suspension, transferred to 15 cm culture dishes (100 g per dish), and incubated at 30 °C with 90–95% relative humidity for 3 days, with periodic mixing. The resulting *Fuqu* samples were then dried at 45 °C for 12 h. The enzyme solution was prepared following the method of Yu et al. [55]. Various enzyme activities were then determined using the following established methods: Glucoamylase activity was determined following the method described by Miller et al. [56]. Amylase activity was assessed following the method described by Gupta et al. [57]. Acidic protease activity was measured according to the method of Zhang et al. [58]. Esterase activity was determined following an adapted procedure from Gilham and Lehner [59], involving adding 4 mL of PVA-olive oil emulsion and 5 mL of 0.025 mol/L KH_2_PO_4_-Na_2_HPO_4_ buffer (pH 7.5) to an Erlenmeyer flask. After adding 1 mL of enzyme solution, the mixture was incubated for 15 min. Following incubation, 15 mL of 95% ethanol and two drops of phenolphthalein were added, and the solution was titrated with 0.05 mol/L NaOH.

### 2.6. Laboratory-Scale Brewing of Sweet Rice Wine

*R. arrhizus* was inoculated onto pre-warmed PDA plates and incubated at 28 °C for 72 h. Spores were scraped into a 50 mL centrifuge tube containing 20 mL of 0.85% sterile NaCl solution. The spore suspension was vortexed with 5 mm glass beads and filtered through gauze. The spore concentration was adjusted to 10^6^ spores/mL using 0.85% sterile NaCl and measured with a haemocytometer [60]. For brewing, 50 g of long-grain glutinous rice and 50 g of distilled water were added to 250 mL Erlenmeyer flasks and sterilised at 115 °C for 30 min. After cooling, the flasks were inoculated with an *R. arrhizus* spore suspension at a concentration of 10^7^ spores/g dry rice and incubated at 30 °C for 48 h. Sterilised water (50 g) was then added to each flask, and fermentation continued for another 72 h. Upon completion of fermentation, the samples were immediately filtered through a 200-mesh nylon filter to obtain the rice wine, which was stored at 4 °C for further analysis.

### 2.7. Determination of Physicochemical Properties, Bioactive Compounds Content, and Antioxidant Capacity

#### 2.7.1. Physicochemical Properties

The total sugar content in the centrifuged fermentation supernatant was measured using a refractometer (Deli, Shanghai, China). Reducing sugars were quantified in the supernatant using the DNS method [56]. Briefly, 0.2 mL of the centrifuged fermentation supernatant filtrate was mixed with 1.8 mL of distilled water in test tubes, followed by shaking. The reducing sugar content was then determined from a standard curve derived from the DNS method. The non-reducing oligosaccharide content was calculated by subtracting the reducing sugar content from the total sugar content [61].

The pH was measured using a Mettler Toledo digital pH meter, calibrated with pH 6.86 and pH 4.00 buffer solutions in accordance with the manufacturer’s instructions. Total acid content was quantified according to the Chinese National Standard for Rice Wine (Huangjiu) (GB/T 13662-2018) protocol [62]. For this analysis, 10 mL of supernatant was mixed with 50 mL of carbon dioxide-free water in a 100 mL beaker and titrated with 0.1 mol/L sodium hydroxide solution until a pH of 8.20 was achieved. Subsequently, 10 mL of 38% formaldehyde was added, and titration was continued with 0.1 mol/L sodium hydroxide solution until a pH of 9.20 was reached to determine the amino nitrogen content.

#### 2.7.2. Bioactive Compounds Content

Bioactive compounds in the sample were analysed using detection kits provided by Beijing Boxbio Science & Technology Co., Ltd.,Beijing, China, in accordance with the manufacturer’s protocols. Total phenolic content was quantified using the Plant Total Phenol Content Detection Kit (AKPL016M, Beijing Boxbio Science & Technology Co., Ltd., Beijing, China.), which is based on the reduction of phosphomolybdic-phosphotungstic acid by phenolic compounds, forming a blue compound with an absorption peak at 760 nm. For this assay, 10 µL of the centrifuged supernatant was mixed with 50 µL of tungstomolybdic acid, 50 µL of alkaline buffer, and 90 µL of distilled water, and the absorbance was measured at 760 nm after incubation.

Total flavonoid content was measured using the Flavonoid Detection Kit (AKPL015M, Beijing Boxbio Science & Technology Co., Ltd., Beijing, China.), which involves the reaction between flavonoids and aluminium ions in an alkaline nitrite solution to form a red complex with an absorption peak at 510 nm. In this procedure, 80 µL of centrifuged supernatant was combined with 20 µL of nitrite solution, followed by the addition of aluminium ion solution, alkaline buffer, and chromogenic solution, with absorbance measured at 510 nm after colour development.

Peptide content was quantified by precipitating proteins from the centrifuged supernatant using 0.4 mol/L TCA (1:3 *v*/*v*). The resulting supernatant was analysed using the BCA Protein Content Detection Kit (AKPR017, Beijing Boxbio Science & Technology Co., Ltd., Beijing, China.), which quantifies peptides based on their ability to reduce Cu^2+^ to Cu^+^ in an alkaline medium, forming a purple complex with the BCA reagent, exhibiting an absorption peak at 562 nm. For this assay, 20 µL of centrifuged supernatant was mixed with 200 µL of BCA working solution and incubated at 37 °C for 30 min. 

Histamine content was determined using the Histamine Content Detection Kit (AKAO026M, Beijing Boxbio Science & Technology Co., Ltd., Beijing, China.) based on the degradation of histamine by histamine dehydrogenase (HDH). In the presence of 1-mPMS, electrons are transferred through WST to produce a soluble formazan product with an absorption peak at 470 nm. The assay involved mixing 30 µL of centrifuged supernatant with buffer, enzyme, and chromogenic agent, followed by absorbance measurement at 470 nm after incubation.

#### 2.7.3. Antioxidant Capacity

The antioxidant capacities of the sample were analysed using detection kits supplied by Beijing Boxbio Science & Technology Co., Ltd., Beijing, China, in accordance with the manufacturer’s protocols. The ABTS assay was performed using the ABTS Free Radical Scavenging Ability Assay Kit (AKAO021M, Beijing Boxbio Science & Technology Co., Ltd., Beijing, China.), which quantifies the ability of antioxidants to scavenge ABTS free radicals. The assay principle is based on the oxidation of ABTS, forming a stable blue-green cation with a characteristic absorption peak at 405 nm. In the presence of antioxidants, ABTS free radicals are neutralised, resulting in a decrease in absorbance, which reflects the radical scavenging capacity of the sample. For the assay, 10 µL of the centrifuged supernatant was mixed with 20 µL of oxidising agent, followed by 170 µL of ABTS working solution. The mixture was incubated at 25 °C for 6 min, and absorbance was measured at 405 nm.

The total antioxidant capacity (T-AOC) of the sample was determined using the Total Antioxidant Capacity (T-AOC) Assay Kit (AKAO012M, Beijing Boxbio Science & Technology Co., Ltd., Beijing, China.), based on the FRAP (Ferric Reducing Ability of Plasma) method. In this assay, antioxidants reduce Fe^3+^-TPTZ to form a blue Fe^2+^-TPTZ complex under acidic conditions, which exhibits a characteristic absorption peak at 593 nm. The change in absorbance reflects the reducing ability of the sample, indicating its total antioxidant capacity. For this assay, 20 µL of the centrifuged supernatant was mixed with 180 µL of FRAP working solution, incubated at 25 °C for 10 min, and absorbance was measured at 593 nm.

### 2.8. Determination of Flavour Compounds

Alcohol content was analysed by gas chromatography following distillation [63]. The glycerol content was quantified using the G0912W kit (Suzhou Grace Biotechnology Co., Ltd., Suzhou, China) in accordance with the manufacturer’s instructions. In this method, glycerol is catalysed by glycerol kinase (GK) to generate glycerol-1-phosphate (G-1-P), which is subsequently oxidised by glycerol phosphate oxidase (GPO) to yield hydrogen peroxide (H_2_O_2_). The H_2_O_2_ reacts with 4-aminoantipyrine to form a red quinone compound, which exhibits a characteristic absorption peak at 510 nm. The glycerol content was determined by measuring the absorbance at this wavelength. Organic acids and amino acids were analysed on an Agilent 1260 II HPLC system (Agilent Technologies, Inc., Santa Clara, CA, USA.). Organic acids were quantified following previously reported methods [64], while amino acids were derivatised with o-phthalaldehyde and 9-fluorenylmethylchloroformate, according to established protocols [65]. Volatile compounds in rice wine samples were extracted by HS-SPME and analysed using GC-MS (Agilent 7890B GC-5977B MSD, Agilent Technologies, Inc., Santa Clara, CA, USA.), following established methods, with injection and GC-MS conditions detailed in a previous report [24]. Volatile compounds were identified by comparing retention times and mass spectra with those of pure standards. Quantification was performed using octyl propionate and L-menthol as internal standards. To assess the contribution of different chemical compounds to the aroma of rice wine, the relative odour activity values (ROAVs) were calculated as the ratio of the compound concentration to its odour threshold, with threshold values obtained from the literature [2,66,67].

### 2.9. Statistical Analysis

All statistical analyses were conducted using GraphPad Prism 9.5.0 (GraphPad Software Inc., San Diego, CA, USA), Origin 2024 (OriginLab Corp., Northampton, MA, USA), and IBM SPSS Statistics 25.0 (SPSS Inc., Chicago, IL, USA). A heatmap was generated to illustrate the volatile compound profiles of different rice wine samples using Chiplot Tools (Version 1.0, accessed on 9 September 2024, https://www.chiplot.online) after Z-score standardisation. Each sample was analysed in triplicate.

## 3. Results and Discussion

### 3.1. Isolation and Identification of Rhizopus

**Figure 3:**
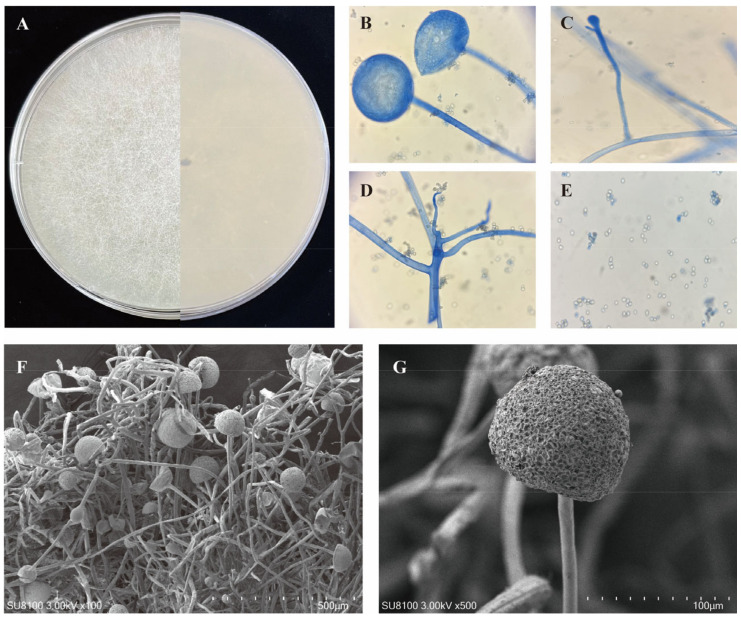
Macroscopic and microscopic morphology of typical *R. arrhizus* (W17) on MEA after 3 days of incubation at 30 °C (**A**); sporangium (**B**); Sporangiophore; Columella, Collarette, Apophysis and Stolon (**C**); Hypha and rhizoids (**D**); spores (**E**); *R. arrhizus* showing a panoramic view of the hyphal and spore structure, magnified at 100×. The dense network of mycelium intertwined with sporangia is evident (**F**); close-up SEM of a *R. arrhizus* sporangium, magnified at 500×, and this detailed view captures the textured surface of the sporangium (**G**).

A total of 78 *Rhizopus* strains were isolated from *Jiuqu* samples collected across 29 regions in China (Supplementary Table S1). These regions were selected for their distinctive fermentation conditions and traditional practises, which reflect the diverse environments of rice wine production. This diversity makes *Jiuqu* an excellent source for isolating autochthonous *R. arrhizus* strains. Subsequently, these strains were identified as *R. arrhizus* through morphological examination on PDA medium and partial ITS region analysis. The morphological examination provided initial identification based on observable traits, such as sporangium shape and hyphal branching. Molecular confirmation was achieved through ITS region analysis, ensuring accurate strain classification [68]. Phylogenetic analysis further elucidated the relationship between the isolated *R. arrhizus* strains and the reference type strain. The typical morphology of *R. arrhizus* (e.g., W17) is depicted in Figure 3, highlighting hyphae, sporangia, and spore structures. Results demonstrated that *Jiuqu* was an effective source for collecting *R. arrhizus* strains, which significantly contributed to the understanding of *R. arrhizus* diversity and adaptability [69]. Morphological and phylogenetic analyses revealed substantial intraspecific variability, supporting the ability of these autochthonous strains to thrive across diverse environments.

### 3.2. Sporulation Ability and Phylogenetic analyses

#### 3.2.1. Sporulation Ability

**Figure 4:**
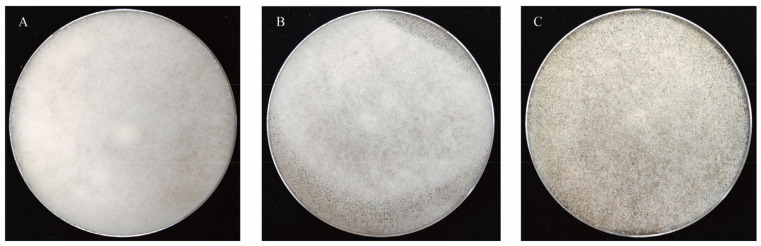
Strains graded according to different levels (**A**–**C**) of sporulation. The complete classification of strains based on sporulation levels is provided in Figure S1.

Three distinct levels of sporulation among *R. arrhizus* strains were identified and categorised as Grades A (low sporulation), B (medium sporulation), and C (high sporulation) (Figure 4). Significant variability in spore production was observed among the 78 strains analysed. All *R. arrhizus* strains used in this study were obtained from *Jiuqu* and are considered autochthonous. These strains exhibited diverse sporulation capacities, indicating significant intraspecific diversity. This variability reflects the inherent genetic and phenotypic diversity of *R. arrhizus* strains traditionally used in fermentation processes. Similar findings have been reported in studies on *Rhizopus microsporus*, where the source of isolation was not significantly associated with molecular differences or reduced sporulation abilities. Moreover, the assumption that specific morphologies, such as those of *Rhizopus oligosporus*, represent separate species confined exclusively to fermented food sources has been contested [52]. These observations underscore the complexity of *Rhizopus* species diversity and the significant role of prolonged domestication in influencing strain characteristics.

#### 3.2.2. Phylogenetic Tree

**Figure 5:**
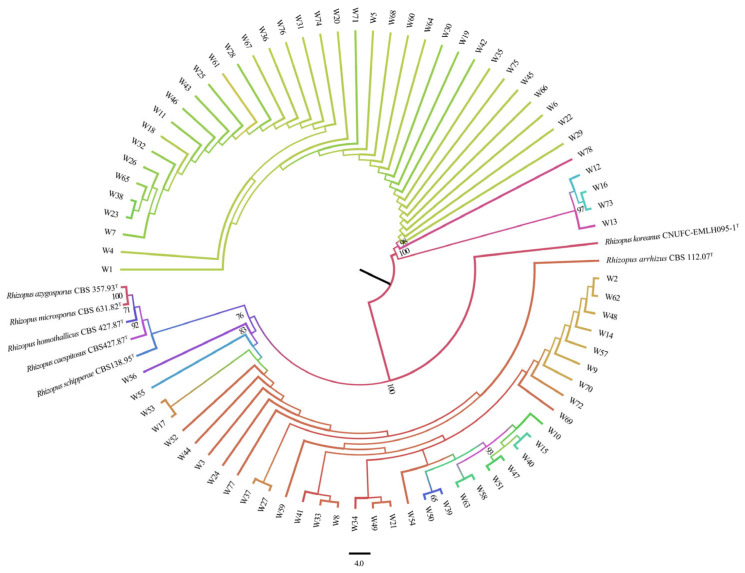
Phylogenetic tree of 78 *R. arrhizus* strains. A phylogenetic tree illustrating the relationships among 78 *R. arrhizus* strains and related species was constructed using ITS sequences and the maximum likelihood (ML) method. Sequence alignment was performed with MAFFT (v7.490). The evolutionary model (HKY+F+G4) was selected using IQ-TREE2 (v2.0.7) with ModelFinder, and phylogenetic analysis was conducted with 1000 ultrafast bootstrap replicates to assess branch support. Bootstrap values below 50 were omitted for clarity. The tree was visualised using FigTree (v1.4.4), and final adjustments were made in Inkscape.

A total of 78 *R. arrhizus* strains and 7 additional *Rhizopus* species were included in the phylogenetic analysis (Figure 5) to evaluate their genetic diversity and phylogenetic relationships. The results demonstrated significant genetic diversity among the *R. arrhizus* strains. Strains W29 and W78 exhibited short genetic distances, indicating a close genetic relationship [70], possibly due to similar genetic backgrounds or ecological adaptations. In contrast, strain W56 was positioned at the bottom of the phylogenetic tree, while strain W1 was located at the top, suggesting a significant genetic distance and intraspecific diversity, which could be attributed to different geographic distributions or ecological environments [70]. Other strains were distributed between these extremes, displaying varying evolutionary distances, indicating a diverse evolutionary history and distinct adaptation mechanisms [71,72]. These genetic differences suggest potential for selecting strains with specific industrial applications.

### 3.3. Enzyme Activity of Different R. arrhizus

**Figure 6:**
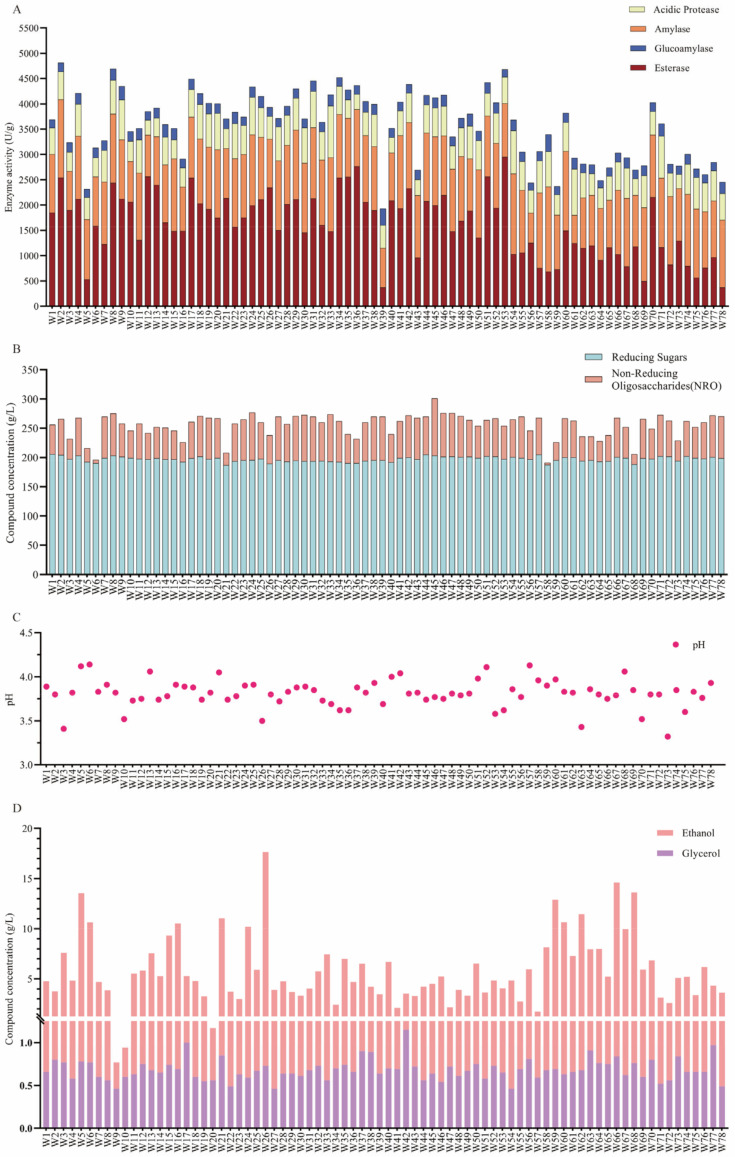
Enzyme activities in 78 strains of *R. arrhizus* (**A**); carbohydrate metabolism capabilities in 78 strains of *R. arrhizus* (**B**); comparison of pH in different *R. arrhizus* fermentations (**C**); ethanol and glycerol production by different *R. arrhizus* strains during fermentation (**D**).

The activities of acid protease, amylase, glucoamylase, and esterase were measured in 78 autochthonous *R. arrhizus* (Figure 6A). Significant differences were observed in acid protease, amylase, and esterase activities among the strains, while glucoamylase activity remained relatively stable. Acid protease activity varied considerably, ranging from 280 U/g to 1023 U/g, with an average of 550 U/g, indicating substantial variation across strains. Yu et al. screened wheat koji for high enzyme activity and identified an *R. arrhizus* strain with elevated acid protease and amylase activities in Huangjiu production, consistent with the findings of this study [55]. These results support the conclusion that rational strain selection can significantly enhance fermentation performance.

Glucoamylase activity was more consistent, averaging 196 U/g, with a range from 148 U/g to 340 U/g, suggesting minimal variation in saccharification potential. Yuan et al. investigated various mould strains in rice wine production and found that strain YM-16 exhibited high glucoamylase and protease activities, similar to the strains screened here [73]. Yang et al. explored the effects of adding specific enzymes during Chinese rice wine fermentation, revealing that supplementation with α-amylase, glucoamylase, and acid protease significantly increased ethanol content, free amino acids, and volatile flavour compounds. This aligns with the high enzyme activity observed in the *R. arrhizus* strains identified in this study, further underscoring the critical role of enzyme activity in enhancing product quality [74]. 

Amylase activity exhibited a wide range, from 557 U/g to 1681 U/g, with an average of 1205 U/g, indicating high variability. Esterase activity spanned from 370 U/g to 2949 U/g, with an average of 1620 U/g, demonstrating significant differences among the strains. Chen et al. significantly improved the aroma and flavour profile of rice wine using mixed fermentation inoculants, highlighting that the high esterase activity *R. arrhizus* strains identified here also have potential for enhancing flavour compound accumulation, further underscoring the importance of high-enzyme strains in improving product quality [75]. 

In summary, the findings of this study align with other research, indicating that selecting and optimising high-enzyme activity strains can enhance fermentation efficiency and improve product flavour quality, providing a scientific basis for further optimisation of brewing processes. The uniqueness of this study lies in its systematic screening of autochthonous *R. arrhizus* strains, particularly for esterase and glucoamylase activities, showcasing their potential for improving the quality and flavour of fermented products.

### 3.4. Physicochemical Properties Analysis

#### 3.4.1. Carbohydrate Metabolism

The sugar content of glutinous rice fermented by 78 autochthonous *R. arrhizus* was measured and statistically analysed. The results showed that the concentration of reducing sugars ranged from 187 to 206 g/L, with minimal variation, and an average of 197 g/L (Figure 6B). Similar studies have demonstrated that specific fermentation strains play a crucial role in regulating sugar levels and fermentation products. For instance, research on black glutinous rice wine revealed that inoculation with particular strains significantly increased sugar utilisation and alcohol yield, thereby shortening the fermentation cycle and enhancing product quality [43]. Additionally, the use of *Jiuqu* from different geographic regions, such as the Sichuan Basin, the coastal plains of Zhejiang, and the Yunnan Plateau, significantly impacted sugar levels and flavour characteristics during fermentation, primarily due to variations in the autochthonous microbial communities adapted to these specific environments [76].

The concentration of oligosaccharides ranged from 4 to 98 g/L, with an average of 58 g/L, indicating substantial differences among *Rhizopus* strains in oligosaccharide production. The trends in reducing sugar and oligosaccharide concentrations further support this finding, with some strains exhibiting marked differences in oligosaccharide production capacity. This aligns with the previous literature, where different strains show significant variation in glucoamylase and amylase expression. For example, certain enhanced *Jiuqu* demonstrated higher amylase activity during fermentation, resulting in increased oligosaccharide production and other flavour compounds [77]. Furthermore, in traditional Korean fermentation rice wine, inoculation with specific strains like *R. arrhizus* significantly elevated the content of soluble solids and reducing sugars, thereby improving the quality and flavour of the fermented wine [47].

In conclusion, *R. arrhizus* consistently produces high concentrations of reducing sugars during fermentation, while oligosaccharide production exhibits greater variability due to strain-specific differences. This variability may be attributed to differences in enzyme activity and metabolic pathways, suggesting that further optimisation is possible in strain selection for brewing applications.

#### 3.4.2. pH, Total Acid, Amino Nitrogen, and Total Peptide

A systematic analysis was conducted on the fermentation products of glutinous rice fermented by 78 autochthonous *R. arrhizus* (Figure 6C), revealing significant differences in pH, total acid, amino nitrogen, and total peptide content. The pH ranged from 3.32 to 4.14, with an average of 3.81. It was observed that substantial acidic compounds were produced during fermentation, primarily as a result of organic acid accumulation, which influenced the pH of the fermentation environment and subsequently affected the formation of other metabolites. This is consistent with previous studies, where *R. arrhizus* was shown to reduce pH through organic acid accumulation during fermentation [78].

**Figure 7:**
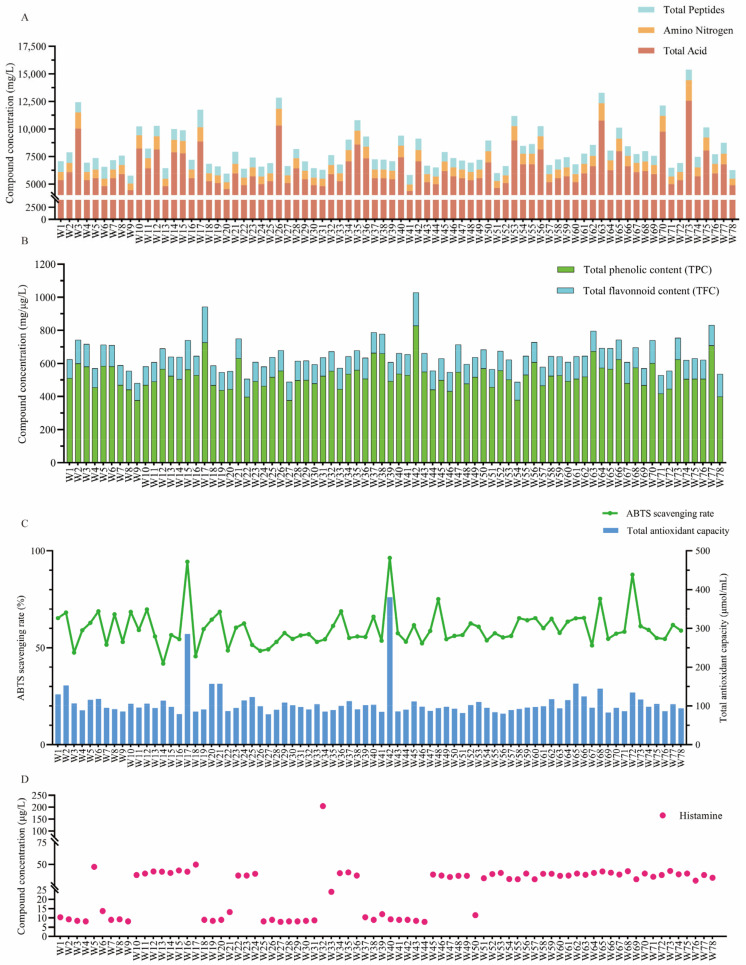
Comparison of total acid, amino nitrogen, and total peptide content in different *R. arrhizus* fermentations (**A**); comparison of total flavonoid content, total phenolic content in different *R. arrhizus* fermentations (**B**); comparison of ABTS scavenging rate, total antioxidant capacity in different *R. arrhizus* fermentations (**C**); comparison of histamine content in different *R. arrhizus* fermentations (**D**).

Total acid content was reported to vary significantly across strains, reflecting substantial differences in acid production capacity (Figure 7A). Total acid content ranged from 2450 to 12,550 mg/L, with an average of 6315 mg/L. It was suggested that this fluctuation could be attributed to differences in metabolic pathways, particularly in the generation and accumulation of organic acids, with some strains demonstrating higher metabolic efficiency. This observation is consistent with studies on red koji rice wine fermentation, where the role of lactic acid bacteria and other microorganisms in organic acid production has been widely recognised [79].

Amino nitrogen content was found to range from 582 to 1870 mg/L, with an average of 899 mg/L, suggesting that some strains exhibited strong protein degradation abilities, releasing more amino acids (Figure 7A). Microbial proteolytic activity has been confirmed to contribute to amino acid accumulation during rice wine brewing in recent studies [61]. This observation is in line with the strong proteolytic abilities observed in *R. arrhizus* strains in this study, highlighting their critical role in amino nitrogen accumulation. Strains W17, W34, and W56 were found to exhibit the highest amino nitrogen content, indicating their superior protein degradation capabilities. These high amino nitrogen strains were suggested to be linked to elevated protease activity, which provided abundant nutrients and improved fermentation quality [80]. This finding is consistent with the significant impact of different starter microbial communities on metabolite production in black glutinous rice wine [81].

Total peptide content was also found to vary considerably, ranging from 705 to 1590 mg/L, with an average of 905 mg/L, indicating differences in the degree of protein hydrolysis among strains (Figure 7A). According to the literature, polypeptides in fermented wine have been shown to play a crucial role in enhancing both flavour and mouthfeel in rice wine, with specific polypeptides interacting synergistically with volatile compounds to significantly improve the sensory quality of the wine [82]. Strains with higher total peptide contents may partially hydrolyze proteins, retaining longer polypeptide chains, which positively influence the flavour, mouthfeel, and complexity of the final product [46]. The generation of total peptides may be influenced by the balance between endopeptidase and exopeptidase activities, leading to polypeptides with specific flavours.

The findings suggest that the 78 autochthonous strains of *R. arrhizus* exhibited a wide range of values for pH, total acid, amino nitrogen, and total peptide content in the fermentation products, indicating their adaptability to diverse rice wine market needs and demonstrating excellent fermentation potential.

#### 3.4.3. Ethanol and Glycerol Content

The ethanol and glycerol content produced by 78 autochthonous *R. arrhizus*-fermented glutinous rice was analysed (Figure 6D). Ethanol concentrations ranged from 0.3 to 16.9 g/L, with an average of 5.2 g/L. The highest ethanol levels, up to 16.9 g/L (2.14% vol), were produced by strains W4, W20, and W61, indicating potential for alcohol production and highlighting substantial variation in ethanol output among the strains. Glycerol concentrations ranged from 0.5 to 1.2 g/L, with an average of 0.7 g/L, showing relatively low variability across strains. It was suggested that glycerol production remained generally stable, likely due to the osmotic regulation mechanisms of the strains. The observed variation in ethanol production was found to reflect differences in the metabolic potential of the strains. These findings are consistent with previous research on *R. arrhizus*, where significant differences in ethanol production capacities among *R. arrhizus* strains from different geographic regions and variants were observed [34,83]. In the production of low-alcohol rice wine, ethanol-producing *Rhizopus* strains could be used to eliminate the need for additional brewing yeast, thereby reducing production costs. Additionally, in the production of dry yellow wine, these strains were found to complete saccharification more efficiently, improving production efficiency. Glycerol accounted for only 0.36% of the fermentation products of *R. arrhizus*, and although some variation in glycerol production among variants was noted, it remained relatively stable across strains [34]. The stability of glycerol production has been attributed to its role as an osmotic regulator during fermentation, consistent with co-fermentation studies involving *R. arrhizus* and fumaric acid [84]. This mechanism may explain the limited variability in glycerol production among strains. 

### 3.5. Bioactive Compounds Content and Antioxidant Capacity

#### 3.5.1. Total Flavonoid Content (TFC) and Total Phenolic Content (TPC)

The TPC and TFC in the fermentation samples of 78 autochthonous *R. arrhizus* strains were measured and analysed (Figure 7B). TPC ranged from 377 to 828 mg/L, with an average of 524 mg/L, indicating significant variation in phenolic compound production during the fermentation of glutinous rice by different *R. arrhizus* strains. Similarly, TFC ranged from 103 to 215 μg/L, with an average of 123 μg/L, reflecting variability in flavonoid synthesis among the strains. Certain strains (e.g., W17 and W42) exhibited higher levels of TPC and TFC, while others (e.g., W9, W27, W54, W70, and W78) displayed lower levels. Studies have also demonstrated that solid-state fermentation with *Rhizopus* can effectively enhance phenolic and flavonoid content. For instance, *R. arrhizus* HC-1 was used by Chen et al. for the solid-state fermentation of soybeans, which significantly increased both TPC and TFC [85]. Similarly, increased phenolic and flavonoid content was observed when wild turmeric was fermented by *R. oligosporus* [86]. Ju et al. reported that pre-treatment of Hibiscus flowers with *R. arrhizus* enhanced flavonoid extraction efficiency by 39.25% due to structural changes in the samples [87]. Silva et al. found that optimal fermentation conditions of brewer’s spent grain with *R. arrhizus* significantly boosted the release of phenolic compounds [88]. Additionally, an increase in phenolic content and alterations in chemical composition were reported by Šelo et al. when grape pomace was fermented with *R. arrhizus* [89]. In conclusion, the results of this study highlight significant differences in phenolic and flavonoid synthesis among *R. arrhizus* strains during glutinous rice fermentation. Strains with higher TPC and TFC have the potential to improve the functional properties and flavour of fermented products and may be further screened for application as superior strains.

#### 3.5.2. ABTS Scavenging Rate and Total Antioxidant Capacity

The ABTS scavenging rate and total antioxidant capacity of 78 autochthonous *R. arrhizus* strains were analysed (Figure 7C). The ABTS scavenging rate ranged from 41.9% to 96.3%, with an average of 60.0%, indicating substantial variability in antioxidant activity among the autochthonous strains. Strains such as W17 and W42 exhibited significantly higher scavenging rates, reaching 96.3%, suggesting strong free-radical scavenging capabilities. These superior rates are likely due to elevated levels of bioactive substances, including peptides, flavonoids, and phenolics. In contrast, some strains demonstrated lower scavenging rates, which may reflect weaker antioxidant activity attributed to physiological differences or variations in antioxidant components within their metabolites. Total antioxidant capacity ranged from 79 to 381 μmol/mL, with an average of 108 μmol/mL, further highlighting significant variation among the strains. Notably, strains W17 and W42 exhibited both higher scavenging rates and total antioxidant capacities, emphasising their potential antioxidant effects through multiple mechanisms.

Solid-state fermentation has been shown to significantly enhance antioxidant activity in various plant matrices. For example, *R. arrhizus* HC-1 used in the solid-state fermentation of soybeans markedly increased total phenolic and flavonoid content, thereby enhancing ABTS scavenging capacity [85]. Similarly, the fermentation of okra by *R. arrhizus* improved the extraction of polyphenolic compounds and significantly increased ABTS radical scavenging activity. Fermented samples consistently demonstrated higher ABTS scavenging rates than unfermented samples, confirming the role of fermentation in enhancing antioxidant properties [87]. A separate study on rice wine found that wine fermented by *R. delemar* exhibited a superior antioxidant capacity compared to wine fermented by *R. arrhizus*, highlighting strain-dependent differences in antioxidant potential [46].

In this study, the higher antioxidant activities observed in strains W17 and W42 can be attributed to their abundance of bioactive compounds, including peptides, phenolics, and flavonoids. Peptides, as bioactive molecules, play a pivotal role in scavenging ABTS radicals due to their unique structures [90], which often include functional groups such as amino and carboxyl groups. These groups can donate hydrogen atoms or electrons, effectively neutralising free radicals. The peptides in W17 and W42 are likely enriched with amino acids such as tyrosine, tryptophan, and cysteine, which are known for their strong antioxidant properties due to their ability to stabilise radical intermediates [91]. Phenolics and flavonoids, other major contributors to antioxidant potential, contain hydroxyl groups capable of donating hydrogen atoms to ABTS radicals, thereby quenching their reactivity. These bioactive compounds not only enhance ABTS scavenging activity but also contribute to broader antioxidant effects, improving the functional properties of fermented products [92,93]. However, discrepancies between ABTS scavenging rates and total antioxidant capacities were observed in certain strains, such as W3 and W25. While these strains exhibited higher ABTS scavenging rates, they had lower total antioxidant capacities, suggesting that their antioxidant compounds may have greater specificity for ABTS radicals but be less effective against other oxidants [94].

In conclusion, W17 and W42 exhibited superior performance in both ABTS scavenging rate and total antioxidant capacity, making them promising candidates for improving the antioxidant properties of traditional fermented alcoholic products and enhancing product functionality. Overall, significant variation in antioxidant capacity among the 78 *R. arrhizus* strains was observed, providing a scientific foundation for further screening of high-quality brewing strains.

#### 3.5.3. Histamine

A quantitative analysis of histamine content in fermented glutinous rice samples from 78 autochthonous *R. arrhizus* strains was conducted to evaluate the ability of different strains to metabolise biogenic amines during fermentation (Figure 7D). *R. arrhizus* has been closely linked to histamine production in fermentation. It has been demonstrated in several studies that *R. arrhizus*, as a key fermentation microorganism, significantly influences the content of biogenic amines, particularly histamine, in fermented foods through its metabolic activity [95,96,97]. Significant variation in histamine content was observed among the strains, with concentrations ranging from 8 to 205 μg/L and an average of 32 μg/L. During the fermentation of red yeast rice wine and xiaoqu rice wine, multiple biogenic amines, including histamine, are produced by *R. arrhizus* through decarboxylation [96,97].

Most strains produced low levels of histamine; however, strain W32 exhibited exceptionally high histamine accumulation, reaching a concentration of 205 μg/L, significantly exceeding the levels observed in other strains. These findings indicate considerable variation in the ability of *R. arrhizus* strains to metabolise histamine during fermentation. High histamine levels could pose potential risks to food safety, making it essential to select strains with low histamine production to improve the safety and quality of fermented products.

### 3.6. Characterisation of Flavour Compounds

#### 3.6.1. Organic Acid

**Figure 8:**
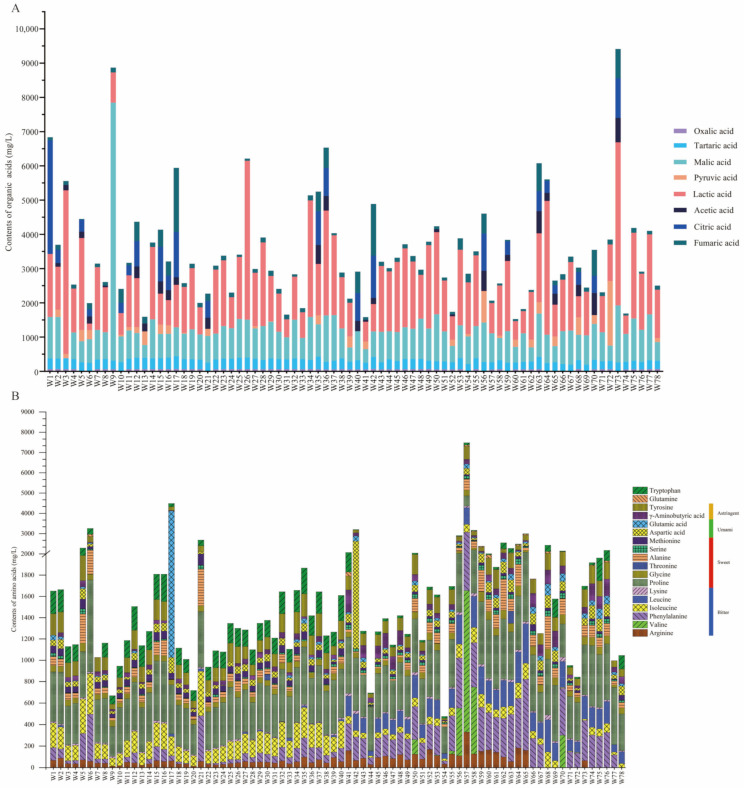
Composition of organic acids (**A**) and amino acids (**B**). (**A**) The stacked bar chart depicts the concentrations of eight organic acids—oxalic acid, tartaric acid, malic acid, pyruvic acid, lactic acid, acetic acid, citric acid, and fumaric acid—in the fermentation samples. Each bar represents the total organic acid content (mg/L) for a specific strain, with individual acids distinguished by colour coding. (**B**) The stacked bar chart illustrates the concentrations of 18 amino acids—tryptophan, glutamine, tyrosine, γ-aminobutyric acid, glutamic acid, aspartic acid, methionine, serine, alanine, threonine, glycine, proline, lysine, leucine, isoleucine, phenylalanine, valine, arginine, and histidine—in the fermentation samples. These amino acids are classified according to their taste profiles (umami, sweet, bitter, or astringent), as indicated by the coloured labels in the legend. The total amino acid content (mg/L) for each strain is represented by a bar, with individual amino acids distinguished by colour coding.

The organic acids produced during the fermentation of glutinous rice by 78 autochthonous *R. arrhizus* strains were systematically analysed (Figure 8A). Significant variation in organic acid production was observed among the strains. *R. arrhizus* is known to play a crucial role in the fermentation of various traditional rice wines and is responsible for generating multiple organic acids [60,76,81,98,99].

The findings indicate that malic acid and lactic acid are the predominant organic acids, with average concentrations constituting 27.22% and 44.51% of the total organic acids, respectively. Fumaric acid and tartaric acid are found to account for 6.54% and 7.88%, while citric acid comprises 6.40%. Pyruvic acid and acetic acid are represented by 3.08% and 2.87%, respectively, with oxalic acid contributing the least at 1.50%. These results suggest that during fermentation, significant production capabilities for lactic acid and malic acid are demonstrated by *R. arrhizus*, with notable accumulation of both acids observed in certain strains. These organic acids are recognised for their essential role in enhancing the acidity, flavour, and overall quality of the wine.

In conclusion, significant differences in organic acid production were identified among autochthonous *R. arrhizus* strains, providing a scientific basis for selecting specific strains to improve fermentation performance.

#### 3.6.2. Amino Acid

The generation of amino acids during the fermentation process by 78 autochthonous strains of *R. arrhizus* strains was systematically analysed (Figure 8B). It was found that glutamic acid and tyrosine were the predominant amino acids in the fermentation samples, with concentration ranges of 0.00–3082.77 mg/L and 16.35–646.02 mg/L, and average concentrations of 65.97 mg/L and 151.60 mg/L, respectively. Significant amounts of L-tyrosine were also produced by *R. arrhizus* AUMC14899, which was isolated from plants. This production not only enhances the flavour of fermented products but also provides antibacterial and anti-biofilm activities [100]. Additionally, it was observed that large quantities of bitter amino acids, such as phenylalanine and arginine, were produced by some strains, with concentrations ranging from 3.30 to 3228.93 mg/L and an average concentration of 528.81 mg/L, which could potentially negatively affect the flavour of the final product.

In studies on red yeast glutinous rice wine, it was demonstrated that the synergistic action of *R. arrhizus* and other microorganisms significantly increased the levels of bitter and umami amino acids [6]. Statistical analysis indicated that the concentration of bitter amino acids ranged from 3.30 to 3228.93 mg/L, with an average of 528.81 mg/L. Sweet amino acids ranged from 0.00 to 538.29 mg/L, with an average of 93.34 mg/L; umami amino acids ranged from 0.00 to 3082.77 mg/L, with an average of 65.97 mg/L; and astringent amino acids ranged from 0.00 to 747.90 mg/L, with an average of 13.92 mg/L. These results suggest that the metabolic profiles of specific strains significantly influence the quality of fermented products, particularly in enhancing umami and flavour. Several strains exhibited significant advantages in the production of multiple amino acids. For example, strain W17 was shown to be particularly efficient in the production of glutamic acid, alanine, and leucine, which are crucial for enhancing the umami and overall flavour of fermented products. Similarly, *R. arrhizus* strain KJJ39, which was isolated from Korean fermenting agents, demonstrated an enhanced effect on amino acid content during rice wine fermentation, with notable accumulations of lysine, leucine, and arginine. These increases not only improved the nutritional value of the rice wine but also enriched its flavour profile [101], resulting in a more robust taste and aroma.

In conclusion, autochthonous *R. arrhizus* strains were found to have a significant capacity to increase amino acid content during fermentation, highlighting their potential to enhance the nutritional value and flavour of various fermented products [47]. By selecting strains that efficiently produce umami and sweet amino acids while minimising bitter amino acids, the overall flavour quality of fermented products can be significantly improved.

#### 3.6.3. Volatile Compounds

**Figure 9:**
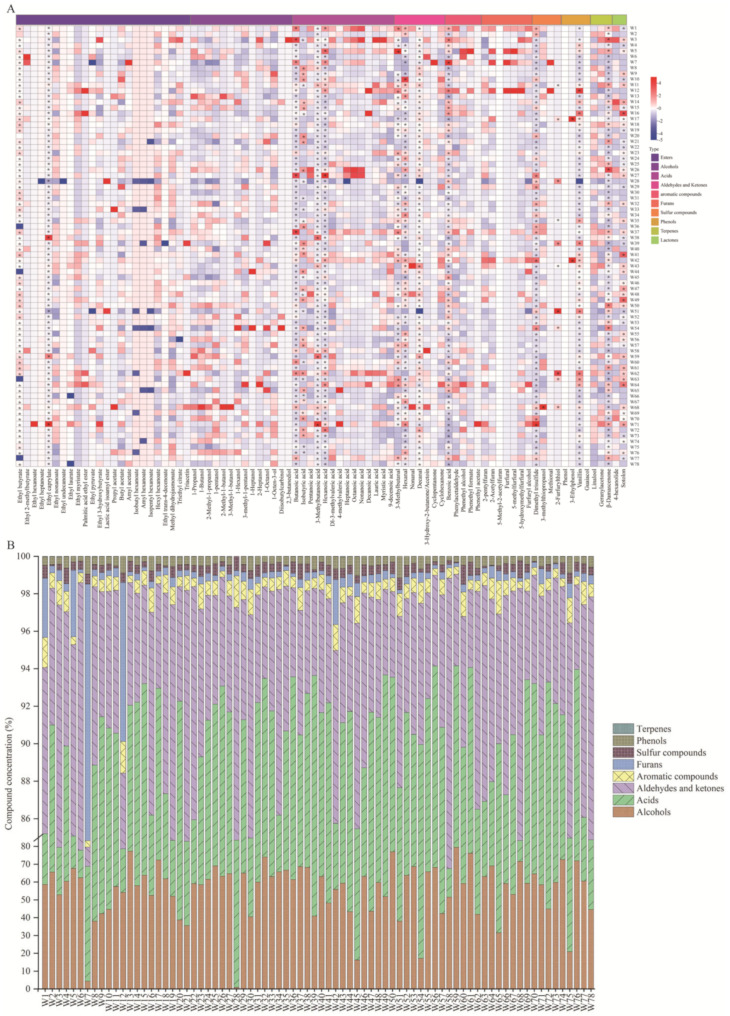
Heatmap visualisation of volatile compounds (**A**); proportions of volatile flavour compounds produced by different *R. arrhizus* strains during fermentation (**B**). (**A**) A heatmap displays the relative concentrations of volatile compounds detected in the fermentation samples, categorised into eight groups: terpenes, phenols, sulphur compounds, furans, aromatic compounds, aldehydes and ketones, acids, and alcohols. These groups are indicated by colour-coded labels at the top of the heatmap. The colour intensity reflects the Z-value, calculated by normalising the compound concentrations across all strains to highlight relative differences. Compounds with ROAV ≥ 1, identified as key aroma-active compounds, are marked with an asterisk (*). (**B**) A stacked bar chart illustrates the proportions of the eight categories of volatile flavour compounds produced by the 78 *R. arrhizus* strains during fermentation. Each bar represents a strain, and the relative abundance (%) of each compound category is shown. Alcohols, acids, and aldehydes and ketones dominate the volatile profiles in most strains, while smaller fractions of esters and sulphur compounds make critical contributions to the overall aroma.

The volatile flavour compounds produced during the fermentation of glutinous rice by 78 autochthonous strains *R. arrhizus* strains were systematically analysed to evaluate their metabolic characteristics and contributions to flavour (Figure 9). 

A total of 80 volatile compounds were identified in the fermentation samples, including esters (24 types), alcohols (14 types), acids (14 types), aldehydes and ketones (7 types), aromatic compounds (5 types), furan compounds (6 types), sulphur-containing compounds (4 types), phenolic compounds (4 types), and terpenes (2 types). Among these, alcohols, acids, aldehydes/ketones, and esters were the most abundant, with average values of 57.83%, 29.85%, 8.36%, and 1.76%. Significant differences in the types and amounts of volatile flavour compounds were evident across the strains. For instance, strain W48 produced higher levels of esters (ethyl butyrate and ethyl caprylate), resulting in a distinct fruity aroma in the fermentation samples, while strain W2 exhibited elevated levels of sulphur-containing compounds, such as dimethyl trisulphide, which enhanced the complexity of the aroma. These differences highlight the diverse metabolic capacities of the strains.

Most ester compounds had relatively low odour thresholds, making them significant contributors to the overall aroma profile of volatile compounds [102]. The concentration and ROAV of these volatile compounds were analysed, identifying 16 key aroma-active compounds with ROAV ≥ 1. These include two esters (ethyl butyrate and ethyl caprylate), four acids (butanoic acid, isobutyric acid, 3-methylbutanoic acid, and hexanoic acid), three aldehydes/ketones (3-methylbutanal, hexanal, and decanal), one aromatic compound (phenylacetaldehyde), three sulphur-containing compounds (dimethyl trisulphide, 3-methylthiopropanol, and 2-furfurylthiol), two phenolic compounds (3-ethylphenol and vanillin), and one terpene (β-damascenone). These compounds, typically formed during autochthonous *R. arrhizus* fermentation, are considered critical in defining the aroma profile of rice wine.

ROAV has been extensively applied in aroma studies of fermented products, including baijiu, yellow wine, and other alcoholic beverages [103,104,105,106], and has been shown to effectively quantify specific aroma characteristics, such as fruity and floral notes. Although gas chromatography–olfactometry and sniffing port analysis were not employed in this study, ROAV calculations are recognised as providing a valuable approximation of the relative contributions of volatile compounds to the overall aroma profile. Matrix effects, such as differences between rice wine and matrices referenced in the literature, are acknowledged as potential influences on odour thresholds. However, previous studies have demonstrated the applicability of these thresholds to similar fermented products. Future research incorporating gas chromatography–olfactometry techniques and matrix-specific threshold measurements will be essential for further validation of these findings.

Among these compounds, 16 key aroma-active compounds were identified, including esters (ethyl butyrate, ethyl caprylate), acids (butanoic acid, isobutyric acid, 3-methylbutanoic acid, hexanoic acid), aldehydes/ketones (3-methylbutanal, hexanal, decanal), an aromatic compound (phenylacetaldehyde), sulphur-containing compounds (dimethyl trisulphide, 3-methylthiopropanol, 2-furfurylthiol), phenolic compounds (3-ethylphenol, vanillin), and a terpene (β-damascenone). These compounds, typically produced during *R. arrhizus* fermentation, are critical in shaping the aroma profile of rice wine.

Regarding ester compounds, ethyl butyrate had an average concentration of 14.02 µg/L, while ethyl isovalerate reached a maximum concentration of 139.25 µg/L, indicating its potential contribution to fruity aromas. Similarly, esters such as butyl acetate and isoamyl butyrate were detected at relatively high average concentrations, contributing fruity and sweet notes to the fermentation samples. Isoamyl butyrate exhibited a high relative odour activity value (ROAV), significantly exceeding the olfactory perception threshold, underscoring its crucial role in fruity aroma formation. Among alcohol compounds, 3-methyl-1-butanol and 1-hexanol were detected at high concentrations, contributing to the complexity and mouthfeel of the fermentation aroma. These findings highlight the importance of metabolic pathways involved in ester and alcohol synthesis in shaping the aromatic profile of fermented products.

For acid compounds, butanoic acid and octanoic acid were detected at higher concentrations in certain strains, significantly influencing the acidity and flavour balance of the fermentation samples. The average concentrations of butanoic acid and octanoic acid were 10.23 µg/L and 1.60 µg/L, respectively, reflecting notable differences in acid metabolism among the strains. Among aldehydes and ketones, 3-methylbutanal exhibited high ROAVs, contributing distinctive fruity notes to the fermentation samples. For aromatic compounds, phenylacetaldehyde, with its high average concentration and ROAV, played an important role in imparting unique spicy and floral aromas to the samples.

Among furan compounds, 2-furfurylthiol exhibited a significant average concentration of 545.41 µg/L, substantially enhancing the complexity of the fermentation aroma. Sulphur-containing compounds, particularly dimethyl trisulphide, showed high ROAVs, contributing distinctive sulphur notes to the aroma profile. For phenolic compounds, 3-ethylphenol and vanillin were detected at higher concentrations in certain strains, imparting smoky and vanilla characteristics to the fermentation samples. 

Other studies have demonstrated that *R. arrhizus* significantly contributes to the production of volatile flavour compounds. In the fermentation of black glutinous rice wine, co-inoculation with *R. arrhizus* and yeast significantly improved wine quality, particularly through enhanced ester synthesis [43]. In sorghum-wine fermentation, the combined action of *R. arrhizus* and acetic acid bacteria promoted the production of alcohol and acid compounds, contributing to the formation of a unique volatile flavour profile [45]. During the fermentation of glutinous rice wine in various regions [73], co-cultivation with *R. arrhizus* significantly increased the concentration of esters, higher alcohols, and organic acids, enhancing the aroma and overall taste harmony [73,107]. Similarly, during sweet rice wine fermentation, *R. arrhizus* improved the production of key flavour compounds, such as ethyl acetate and β-phenylethyl alcohol [46].

In conclusion, different strains of autochthonous *R. arrhizus* produced diverse volatile flavour compounds during fermentation, influencing the aroma and flavour quality of fermented products. By analysing these compounds, strains with favourable aromatic profiles can be identified, offering a basis for selecting optimal brewing strains tailored to specific aroma requirements. 

## 4. Conclusions

In this study, 78 autochthonous strains of *R. arrhizus* were isolated and identified from *Jiuqu* samples collected from 29 regions in China to assess their role in traditional rice wine brewing. Comprehensive analyses were conducted on these strains, including morphological observation, phylogenetic analysis, enzyme activity measurement, and fermentation performance evaluation. Significant morphological and genetic diversity was observed among the strains, with sporulation abilities categorised into low, moderate, and high groups. Intraspecific differences were revealed through phylogenetic analysis. 

Strains W17 and W42 were identified as the most promising candidates due to their exceptional performance across multiple parameters. High enzyme activities (acidic protease, amylase, and esterase) were observed, along with strong antioxidant capacities, demonstrated by an ABTS scavenging rate of 96.3% and a total antioxidant capacity of 381 μmol/mL. Additionally, the production of key bioactive compounds, such as phenolics and flavonoids, was recorded. Furthermore, the aroma and flavour profiles of rice wine were significantly enhanced by these strains through the production of essential volatile compounds, including esters and alcohols. 

This study provides a scientific basis for screening and optimising autochthonous *R. arrhizus* strains originating from China with specific enzyme activities and fermentation properties, offering valuable strain resources for developing synthetic microbial communities. These strains can be applied to enhance rice wine quality, supporting market demand for diversity, personalisation, and functionality in rice wine products. The findings also provide a reference for developing other functional strains and optimising product characteristics.

## Data Availability

The original contributions presented in this study are included within the article. The ITS sequences of the 78 isolated strains of *Rhizopus arrhizus* have been uploaded to NCBI GenBank, with accession numbers provided in Table S1.

## Supplementary Materials

The following supporting information can be downloaded at: https://www.mdpi.com/article/10.3390/foods14020312/s1, Table S1: Original information of involved strains of *Rhizopus arrhizus*; Figure S1: Comprehensive classification of *Rhizopus arrhizus* strains based on sporulation levels (Grades A–C).

## Statement

During the preparation of this work, the authors used ChatGPT-4o with canvas and ChatGPT-o1 in order to improve the readability and language of the manuscript. After using these tools, the authors reviewed and edited the content as needed and take full responsibility for the content of the published article.

## References

[1] Lv R.L., Chantapakul T., Zou M.M., Li M., Zhou J.W., Ding T., Ye X.Q., Liu D.H. (2018). Thermal inactivation kinetics of *Bacillus cereus* in Chinese rice wine and in simulated media based on wine components. Food Control.

[2] Yu H., Guo W., Ai L., Chen C., Tian H. (2022). Unraveling the difference in aroma characteristics of *Huangjiu* from Shaoxing region fermented with different brewing water, using descriptive sensory analysis, comprehensive two-dimensional gas chromatography-quadrupole mass spectrometry and multivariate data analysis. Food Chem..

[3] Zhang J.X., Li T., Zou G., Wei Y.J., Qu L.B. (2024). Advancements and future directions in yellow rice wine production research. Fermentation.

[4] Wang J., Yuan C., Gao X., Kang Y., Huang M., Wu J., Liu Y., Zhang J., Li H., Zhang Y. (2020). Characterization of key aroma compounds in *Huangjiu* from northern China by sensory-directed flavor analysis. Food Res. Int..

[5] Huang Z.R., Guo W.L., Zhou W.B., Li L., Xu J.X., Hong J.L., Liu H.P., Zeng F., Bai W.D., Liu B. (2019). Microbial communities and volatile metabolites in different traditional fermentation starters used for *Hong Qu* glutinous rice wine. Food Res. Int..

[6] Liang Z., Lin X., He Z., Su H., Li W., Ren X. (2020). Amino acid and microbial community dynamics during the fermentation of *Hong Qu* glutinous rice wine. Food Microbiol..

[7] Pan X.D., Tang J., Chen Q., Wu P.G., Han J.L. (2013). Evaluation of direct sampling method for trace elements analysis in Chinese rice wine by ICP-OES. Eur. Food Res. Technol..

[8] Peng L., Ai-Lati A., Ji Z., Chen S., Mao J. (2019). Polyphenols extracted from *huangjiu* have anti-inflammatory activity in lipopolysaccharide stimulated RAW264.7 cells. RSC Adv.

[9] Jeong J.W., Nam P.W., Lee S.J., Lee K.G. (2011). Antioxidant activities of Korean rice wine concentrates. J. Agric. Food Chem..

[10] Lin H., Zhang J., Ni T., Lin N., Meng L., Gao F., Luo H., Liu X., Chi J., Guo H. (2019). Yellow wine polyphenolic compounds prevent doxorubicin-induced cardiotoxicity through activation of the Nrf2 signalling pathway. J. Cell. Mol. Med..

[11] Zhao F., Ji Z., Chi J., Tang W., Zhai X., Meng L., Guo H. (2016). Effects of Chinese yellow wine on nitric oxide synthase and intercellular adhesion molecule-1 expressions in rat vascular endothelial cells. Acta Cardiol..

[12] Luo J., Mills K., le Cessie S., Noordam R., van Heemst D. (2020). Ageing, age-related diseases and oxidative stress: What to do next. Ageing Res. Rev..

[13] Hayes J.D., Dinkova-Kostova A.T., Tew K.D. (2020). Oxidative stress in cancer. Cancer Cell.

[14] Guan R., Van Le Q., Yang H., Zhang D., Gu H., Yang Y., Sonne C., Lam S.S., Zhong J., Jianguang Z. (2021). A review of dietary phytochemicals and their relation to oxidative stress and human diseases. Chemosphere.

[15] Caliceti C., Rizzo P., Giuliano M. (2018). Role of natural compounds in oxidative stress and inflammation linked to cardiometabolic disorders: From biochemical aspects to clinical evidences. Oxidative Med. Cell. Longev..

[16] Rousta N., Aslan M., Yesilcimen Akbas M., Ozcan F., Sar T., Taherzadeh M.J. (2024). Effects of fungal based bioactive compounds on human health: Review paper. Crit. Rev. Food Sci. Nutr..

[17] Zhao C., Su W., Mu Y., Mu Y., Jiang L. (2020). Integrative metagenomics-metabolomics for analyzing the relationship between microorganisms and non-volatile profiles of traditional *Xiaoqu*. Front. Microbiol..

[18] Went F.A.F.C., Prinsen Geerligs H.C. (1895). Beobachtungen über die hefearten und zuckerbildenden pilze der arackfabrikation. Verh. K. Akad. Wet..

[19] Peng Q., Zheng H.J., Meng K., Yu H.F., Xie G.F., Zhang Y.H., Yang X.Y., Chen J.L., Xu Z.Q., Lin Z.C. (2022). Quantitative study on core bacteria producing flavor substances in *Huangjiu* (Chinese yellow rice wine). LWT-Food Sci. Technol..

[20] Hong X., Chen J., Liu L., Wu H., Tan H., Xie G., Xu Q., Zou H., Yu W., Wang L. (2016). Metagenomic sequencing reveals the relationship between microbiota composition and quality of Chinese rice wine. Sci. Rep..

[21] Londono-Hernandez L., Ramirez-Toro C., Ruiz H.A., Ascacio-Valdes J.A., Aguilar-Gonzalez M.A., Rodriguez-Herrera R., Aguilar C.N. (2017). *Rhizopus oryzae*-Ancient microbial resource with importance in modern food industry. Int. J. Food Microbiol..

[22] Dong W., Zeng Y., Ma J., Cai K., Guo T., Tan G., Yu X., Hu Y., Peng N., Zhao S. (2024). Characteristics and functions of dominant yeasts together with their applications during strong-flavor baijiu brewing. Foods.

[23] Fan J., Qu G., Wang D., Chen J., Du G., Fang F. (2023). Synergistic fermentation with functional microorganisms improves safety and quality of traditional Chinese fermented foods. Foods.

[24] Gao R.J., Peng P., Yu L., Wan B., Liang X.T., Liu P.L., Liao W.F., Miao L.H. (2024). Metagenomic analysis reveals the correlations between microbial communities and flavor compounds during the brewing of traditional *Fangxian huangjiu*. Food Biosci..

[25] Yuan Y., Yang Y., Xiao L., Qu L., Zhang X., Wei Y. (2023). Advancing insights into probiotics during vegetable fermentation. Foods.

[26] Zheng R.Y., Chen G.Q., Huang H., Liu X.Y. (2007). A monograph of *Rhizopus*. Sydowia.

[27] Zhao H., Nie Y., Zong T.K., Wang K., Lv M.L., Cui Y.J., Tohtirjap A., Chen J.J., Zhao C.L., Wu F. (2023). Species diversity, updated classification and divergence times of the phylum Mucoromycota. Fungal Divers..

[28] Liu X.Y., Huang H., Zheng R.Y. (2008). Delimitation of *Rhizopus* varieties based on IGS rDNA sequences. Sydowia.

[29] Dolatabadi S., de Hoog G.S., Meis J.F., Walther G. (2014). Species boundaries and nomenclature of *Rhizopus arrhizus* (syn. *R. oryzae*). Mycoses.

[30] Saito K., Saito A., Ohnishi M., Oda Y. (2004). Genetic diversity in *Rhizopus oryzae* strains as revealed by the sequence of lactate dehydrogenase genes. Arch. Microbiol..

[31] Yao L.D., Ju X., James T.Y., Qiu J.Z., Liu X.Y. (2018). Relationship between saccharifying capacity and isolation sources for strains of the *Rhizopus arrhizus* complex. Mycoscience.

[32] Ju X., Zhang M.Z., Zhao H., Liu Z., Jia B.S., Timothy Y.J., Qiao M., Liu X.R. (2020). Genomic SNPs reveal population structure of *Rhizopus arrhizus*. Mycosystema.

[33] Yao L.D., Ju X., Timothy Y.J., Liu X.Y., Qiu J.Z. (2019). Diversity of growth kinetic models for *Rhizopus arrhizus*. Microbiol. China.

[34] Liu X.L., Ju X., Jia B.S., Timothy Y.J., Qiao M., Liu X.Y. (2022). The correlations of fermentation metabolites with sporulation capability and varieties of *Rhizopus arrhizus*. Acta Microbiol. Sin..

[35] Wu H., Liu H.N., Ma A.M., Zhou J.Z., Xia X.D. (2022). Synergetic effects of *Lactobacillus plantarum* and *Rhizopus oryzae* on physicochemical, nutritional and antioxidant properties of whole-grain oats (*Avena sativa* L.) during solid-state fermentation. LWT-Food Sci. Technol..

[36] Cantabrana I., Perise R., Hernández I. (2015). Uses of *Rhizopus oryzae* in the kitchen. Int. J. Gastron. Food Sci..

[37] Araujo L.P., Vilela H., Solinho J., Pinheiro R., Belo I., Lopes M. (2024). Enrichment of fruit peels’ nutritional value by solid-state fermentation with *Aspergillus ibericus* and *Rhizopus oryzae*. Molecules.

[38] Drabo M.S., Savadogo A., Raes K. (2023). Effects of tempeh fermentation using *Rhizopus oryzae* on the nutritional and flour technological properties of *Zamnè* (*Senegalia macrostachya* seeds): Exploration of processing alternatives for a hard-to-cook but promising wild legume. Food Biosci..

[39] Balogun O.I., Epriliati I., Otunola E.T., Agboola H.A. (2017). *Rhizopus oryzae* FNCC 6010, *Rhizopus oligosporus* FNCC 6011, and their hybrid lowered antioxidant capacity in *velvet beans* compared to germination. Int. Food Res. J..

[40] Zhao H., Ju X., Nie Y., James T.Y., Liu X.Y. (2024). High-throughput screening carbon and nitrogen sources to promote growth and sporulation in *Rhizopus arrhizus*. AMB Express.

[41] Ibarruri J., Cebrián M., Hernández I. (2019). Solid state fermentation of brewer’s spent grain using *Rhizopus sp*. to enhance nutritional value. Waste Biomass Valorization.

[42] Mu Y., Huang Y., Li D., Zhu Z., Yu S., Xie F. (2024). Revealing the comprehensive effect of mechanization on sauce-flavor *Daqu* through high-throughput sequencing and multi-dimensional metabolite profiling. Food Res. Int..

[43] Zhao C., Su W., Mu Y., Luo L., Zhao M., Qiu S., Su G., Jiang L. (2023). Effects of *Jiuqu* inoculating *Rhizopus oryzae* Q303 and *Saccharomyces cerevisiae* on chemical components and microbiota during black glutinous rice wine fermentation. Int. J. Food Microbiol..

[44] Yu H.Y., Li Z.Q., Zheng D.W., Chen C., Ge C., Tian H.X. (2024). Exploring microbial dynamics and metabolic pathways shaping flavor profiles in Huangjiu through metagenomic analysis. Food Res. Int..

[45] Chen L.H., Liu B., Feng S.B., Ma X., Wang S.X., Zhang Y.T. (2023). Correlation between microbe, physicochemical properties of *Jiuqu* in different plateau areas and volatile flavor compounds of highland barley alcoholic drink. Food Biosci..

[46] Yang Y.R., Zhong H.Y., Yang N., Xu S.Z., Yang T. (2022). Quality improvement of sweet rice wine fermented with *Rhizopus delemar* on key aroma compounds content, phenolic composition, and antioxidant capacity compared to *Rhizopus oryzae*. J. Food Sci. Technol.-Mysore.

[47] Kim M., Seo J.A. (2021). Fermentation profiling of rice wine produced by *Aspergillus oryzae* KSS2 and *Rhizopus oryzae* KJJ39 newly isolated from Korean fermentation starter. Appl. Biol. Chem..

[48] Yu H., Liu S., Qin H., Zhou Z., Zhao H., Zhang S., Mao J. (2024). Artificial intelligence-based approaches for traditional fermented alcoholic beverages’ development: Review and prospect. Crit. Rev. Food Sci. Nutr..

[49] Ma D., Liu S., Liu H., Zhang S., Xu Y., Mao J. (2024). Environmental factors drive microbial community succession in biofortified *wheat Qu* and its improvement on the quality of Chinese *huangjiu*. J. Biosci. Bioeng..

[50] Dong W.W., Yang Q., Liao Y.X., Liu Y.C., Hu Y.L., Peng N., Liang Y.X., Zhao S.M. (2020). Characterisation and comparison of the microflora of traditional and pure culture *xiaoqu* during the *baijiu* liquor brewing process. J. Inst. Brew..

[51] Chen X., Song C., Zhao J., Xiong Z., Peng L., Zou L., Liu B., Li Q. (2024). Effect of a new fermentation strain combination on the fermentation process and quality of highland barley yellow wine. Foods.

[52] Dolatabadi S., Walther G., Gerrits van den Ende A.H.G., Hoog G.S. (2013). Diversity and delimitation of *Rhizopus microsporus*. Fungal Divers..

[53] Hota S., Achary K.G., Singh S. (2023). Identification and molecular characterization of *Rhizopus delemar* from Eastern Ghats of state of India and its biotechnological applications. Geomicrobiol. J..

[54] Minh B.Q., Schmidt H.A., Chernomor O., Schrempf D., Woodhams M.D., von Haeseler A., Lanfear R. (2020). Corrigendum to: IQ-TREE 2: New models and efficient methods for phylogenetic inference in the genomic era. Mol. Biol. Evol..

[55] Yu P., Du J., Cao C., Cai G., Sun J., Wu D., Lu J. (2021). Development of a novel multi-strain wheat Qu with high enzyme activities for Huangjiu fermentation. J. Sci. Food Agric..

[56] Miller G.L. (2002). Use of dinitrosalicylic acid reagent for determination of reducing sugar. Anal. Chem..

[57] Gupta R., Gigras P., Mohapatra H., Goswami V.K., Chauhan B. (2003). Microbial α-amylases: A biotechnological perspective. Process Biochem..

[58] Zhang W.Q., Si G.R., Rao Z.M., Li J.L., Zhang X., Mei J., Wang J.S., Ye M., Zhou P. (2019). High yield of tetramethylpyrazine in functional Fuqu using bacillus amyloliquefaciens. Food Biosci..

[59] Gilham D., Lehner R. (2005). Techniques to measure lipase and esterase activity in vitro. Methods.

[60] Yuan H.W., Zhang C., Chen S.Y., Zhao Y., Tie Y., Yin L.G., Chen J., Wu Q.D., Wang Y.T., Xu Z. (2023). Effect of different moulds on oenological properties and flavor characteristics in rice wine. LWT Food Sci. Technol..

[61] Li J., Tang X., Qian H., Yang Y., Zhu X., Wu Q., Mu Y., Huang Z. (2021). Analysis of saccharification products of high-concentration glutinous rice fermentation by *Rhizopus nigricans* Q3 and alcoholic fermentation of *Saccharomyces cerevisiae* GY-1. ACS Omega.

[62] (2018). Huangjiu.

[63] Liu P., Miao L. (2020). Multiple batches of fermentation promote the formation of functional microbiota in Chinese miscellaneous-flavor baijiu fermentation. Front. Microbiol..

[64] Liu S., Chen Q., Zou H., Yu Y., Zhou Z., Mao J., Zhang S. (2019). A metagenomic analysis of the relationship between microorganisms and flavor development in Shaoxing mechanized huangjiu fermentation mashes. Int. J. Food Microbiol..

[65] Gong M., Zhou Z.L., Yu Y.J., Liu S.P., Zhu S.H., Jian D.Z., Cui P.J., Zhong F., Mao J. (2020). Investigation of the 5-hydroxymethylfurfural and furfural content of Chinese traditional fermented vinegars from different regions and its correlation with the saccharide and amino acid content. LWT Food Sci. Technol..

[66] Wang J., Zhang B., Wu Q., Jiang X., Liu H., Wang C., Huang M., Wu J., Zhang J., Yu Y. (2022). Sensomics-assisted flavor decoding of coarse cereal Huangjiu. Food Chem..

[67] Wang R., Sun J.C., Lassabliere B., Yu B., Liu S.Q. (2022). Green tea fermentation with *Saccharomyces boulardii* CNCM I-745 and *Saccharomyces boulardii* 299V. LWT Food Sci. Technol..

[68] Lucking R., Aime M.C., Robbertse B., Miller A.N., Ariyawansa H.A., Aoki T., Cardinali G., Crous P.W., Druzhinina I.S., Geiser D.M. (2020). Unambiguous identification of fungi: Where do we stand and how accurate and precise is fungal DNA barcoding. IMA Fungus.

[69] Yang Q., Yao H., Liu S., Mao J. (2021). Interaction and application of molds and yeasts in Chinese fermented foods. Front. Microbiol..

[70] Sjamsuridzal W., Khasanah M., Febriani R., Vebliza Y., Oetari A., Santoso I., Gandjar I. (2021). The effect of the use of commercial tempeh starter on the diversity of *Rhizopus tempeh* in Indonesia. Sci. Rep..

[71] Gryganskyi A.P., Golan J., Muszewska A., Idnurm A., Dolatabadi S., Mondo S.J., Kutovenko V.B., Kutovenko V.O., Gajdeczka M.T., Anishchenko I.M. (2023). Sequencing the genomes of the first terrestrial fungal lineages: What have we learned. Microorganisms.

[72] Marsit S., Leducq J.B., Durand E., Marchant A., Filteau M., Landry C.R. (2017). Evolutionary biology through the lens of budding yeast comparative genomics. Nat. Rev. Genet..

[73] Yuan H., Wu Z., Liu H., He X., Liao Z., Luo W., Li L., Yin L., Wu F., Zhang L. (2024). Screening, identification, and characterization of molds for brewing rice wine: Scale-up production in a bioreactor. PLoS ONE.

[74] Yang L., Zhou Y., Li J.L., Liu S., He S.D., Sun H.J., Yao S.F., Xu S.Y. (2021). Effect of enzymes addition on the fermentation of Chinese rice wine using defined fungal starter. LWT-Food Sci. Technol..

[75] Chen L.H., Wang S.X., Ren L.X., Li D.N., Ma X., Rong Y.Z. (2021). Flavour characteristics of rice wine fermented with mixed starter by moulds and yeast strains. Int. J. Food Sci. Tech..

[76] Chen L., Xiang W., Liang X., Liu J., Zhu H., Cai T., Zhang Q., Tang J. (2023). Fungal biomarkers in traditional starter determine the chemical characteristics of turbid rice wine from the rim of the Sichuan Basin, China. Foods.

[77] Zhu Y., Liu S.P., Ma D.L., Xu Y.Z., Yang C., Mao J. (2023). Stabilization of *jiuyao* quality for *huangjiu* brewing by fortifying functional strains based on core microbial community analysis. Food Biosci..

[78] Wu N., Zhang J., Ou W., Chen Y., Wang R., Li K., Sun X.M., Li Y., Xu Q., Huang H. (2021). Transcriptome analysis of *Rhizopus oryzae* seed pellet formation using triethanolamine. Biotechnol. Biofuels Bioprod..

[79] Qian M., Ruan F., Zhao W., Dong H., Bai W., Li X., Huang X., Li Y. (2023). The dynamics of physicochemical properties, microbial community, and flavor metabolites during the fermentation of semi-dry Hakka rice wine and traditional sweet rice wine. Food Chem..

[80] Jin Z., Cai G., Wu C., Hu Z., Xu X., Xie G., Wu D., Lu J. (2021). Profiling the key metabolites produced during the modern brewing process of Chinese rice wine. Food Res. Int..

[81] Jiang L., Su W., Mu Y., Mu Y. (2020). Major metabolites and microbial community of fermented black glutinous rice wine with different starters. Front. Microbiol..

[82] Zhou M., Bu T., Zheng J., Liu L., Yu S., Li S., Wu J. (2021). Peptides in brewed wines: Formation, structure, and function. J. Agric. Food Chem..

[83] Ju X., Yao L.D., Bai Y.C., Qiu J.Z., Liu X.Y. Exploration of alcohol production and alcohol tolerance of *Rhizopus arrhizus*. Proceedings of the 2018 Annual Meeting of Mycological Society of China.

[84] Liu H., Xiao Q., Yue Y., Huang X.L., Zhang Y.K., Deng L., Wang F. (2021). Influence analysis of glycerol in fumaric acid co-fermentation process by *Rhizopus arrhizus*. J. Environ. Chem. Eng..

[85] Chen J.X., Chen Y.l., Hu J.J., He C., Peng X.Z., Li Z.J., Wang Y.L., Zhu M.Z., Xiao Y. (2023). Solid-state fermentation with *Rhizopus oryzae* HC-1 improves the metabolites profiling, antioxidant activity and gut microbiota modulation effect of soybeans. LWT-Food Sci. Technol..

[86] Lim J., Nguyen T.T.H., Pal K., Gil Kang C., Park C., Kim S.W., Kim D. (2022). Phytochemical properties and functional characteristics of wild turmeric (*Curcuma aromatica*) fermented with *Rhizopus oligosporus*. Food Chem. X.

[87] Ju X., Chen T., Ding Y., Yu D., Zhang J., Zhang R., Zhang Y., Wang X., Xu T., Li J. (2024). Effects of *Rhizopus arrhizus* 31-assisted pretreatment on the extraction and bioactivity of total flavonoids from *Hibiscus manihot* L. Molecules.

[88] Silva L.C., Kupski L., Beserra da Silva de Souza S., Bolanho Barros B.C. (2024). Influence of fermentation conditions by *Rhizopus oryzae* on the release of phenolic compounds, composition, and properties of brewer’s spent grain. Food Biosci..

[89] Selo G., Planinic M., Tisma M., Martinovic J., Perkovic G., Bucic-Kojic A. (2023). Bioconversion of grape pomace with *Rhizopus oryzae* under solid-state conditions: Changes in the chemical composition and profile of phenolic compounds. Microorganisms.

[90] He Z., Zhou Y., Li S., Li W., Zhang Y., Guo C., Guo Z., Wei B., Bi Y. (2024). Bioactive Peptides and Evaluation of Cardiac Cytoprotective Effects of Red Millet Yellow Wine as Functional Food. Foods.

[91] Tsuji A., Koyanagi T. (2025). Significant contribution of amino acids to antioxidant capacity of Japanese rice wine (sake). J. Sci. Food Agric..

[92] Ren N., Gong W., Zhao Y., Zhao D.G., Xu Y. (2022). Innovation in *sweet rice wine* with high antioxidant activity: *Eucommia ulmoides* leaf sweet rice wine. Front. Nutr..

[93] Gao Y., Wang Y., Hu L., Wang N., Cui F., Ying S., Hu F. (2024). Research on the brewing technology of *Dangshen Huangjiu* with low biogenic amines and high functional factors. J. Sci. Food Agric..

[94] Xiang Z., Liu S., Qiu J., Lin H., Li D., Jiang J. (2023). Identification and quality evaluation of Chinese rice wine using UPLC-PDA-QTOF/MS with dual-column separation. Phytomedicine.

[95] Qin F.Y., Wu Z.Y., Zhang W.X. (2022). Evaluation of six commercial koji on the formation of biogenic amines and higher alcohols in rice wine. J. Inst. Brew..

[96] Yan Y., Liang Z., Huo Y., Wu Q., Ni L., Lv X. (2024). A comparative study of microbial communities, biogenic amines, and volatile profiles in the brewing process of rice wines with Hongqu and Xiaoqu as fermentation starters. Foods.

[97] Yang Z., Li W., Yuan Y., Liang Z., Yan Y., Chen Y., Ni L., Lv X. (2023). Metagenomic insights into the regulatory effects of microbial community on the formation of biogenic amines and volatile flavor components during the brewing of *Hongqu* rice wine. Foods.

[98] Zhao C., Su W., Mu Y., Jiang L., Mu Y. (2020). Correlations between microbiota with physicochemical properties and volatile flavor components in black glutinous rice wine fermentation. Food Res. Int..

[99] Yu H., Li Q., Guo W., Chen C., Ai L., Tian H. (2023). Dynamic analysis of volatile metabolites and microbial community and their correlations during the fermentation process of traditional *Huangjiu* (Chinese rice wine) produced around winter solstice. Food Chem. X.

[100] El-Zawawy N.A., Ali S.S., Nouh H.S. (2023). Exploring the potential of *Rhizopus oryzae* AUMC14899 as a novel endophytic fungus for the production of L-tyrosine and its biomedical applications. Microb. Cell Fact..

[101] Liang Z.C., Lin X.Z., He Z.G., Su H., Li W.X., Guo Q.Q. (2020). Comparison of microbial communities and amino acid metabolites in different traditional fermentation starters used during the fermentation of Hong Qu glutinous rice wine. Food Res. Int..

[102] Xu Y., Zhao J., Liu X., Zhang C., Zhao Z., Li X., Sun B. (2022). Flavor mystery of Chinese traditional fermented baijiu: The great contribution of ester compounds. Food Chem..

[103] Xu J., Zhang T., Chen H., Dai Y., Li Z., He J., Ju R., Hou A. (2024). Study on the Fermented Grain Characteristics and Volatile Flavor Substances during the *Tuqu* Fermentation of Hunan Light-Flavor Baijiu. Foods.

[104] Li Y., Qiao H., Zhang R., Zhang W., Wen P. (2023). Microbial Diversity and Volatile Flavor Compounds in Tibetan Flavor *Daqu*. Foods.

[105] Xia D., Tan X., Wang L., Li Z., Hou A., Zhu Y., Lai L., Wang Y. (2023). GC-MS Coupled with Rate-All-That-Apply (RATA) to Analyse the Volatile Flavor Substances of *Yellow Wine* during Fermentation. Foods.

[106] Wang J., Wang Z., He F., Pan Z., Du Y., Chen Z., He Y., Sun Y., Li M. (2024). Effect of microbial communities on flavor profile of Hakka rice wine throughout production. Food Chem. X.

[107] Xiang W., Xu Q., Zhang N., Rao Y., Zhu L., Zhang Q. (2019). *Mucor indicus* and *Rhizopus oryzae* co-culture to improve the flavor of Chinese turbid rice wine. J. Sci. Food Agric..

[108] Jiang S., Li H., Zhang L., Mu W., Zhang Y., Chen T., Wu J., Tang H., Zheng S., Liu Y. (2024). Generic diagramming platform (GDP): A comprehensive database of high-quality biomedical graphics. Nucleic Acids Res..

