# Supplementary figures and images for: Isolation and Evaluation of Rhizopus arrhizus Strains from Traditional Rice Wine Starters (Jiuqu): Enzyme Activities, Antioxidant Capacity, and Flavour Compounds
Source: Foods. 2025 Jan 17;14(2):312. doi: 10.3390/foods14020312 (PMC11765298; doi:10.3390/foods14020312)

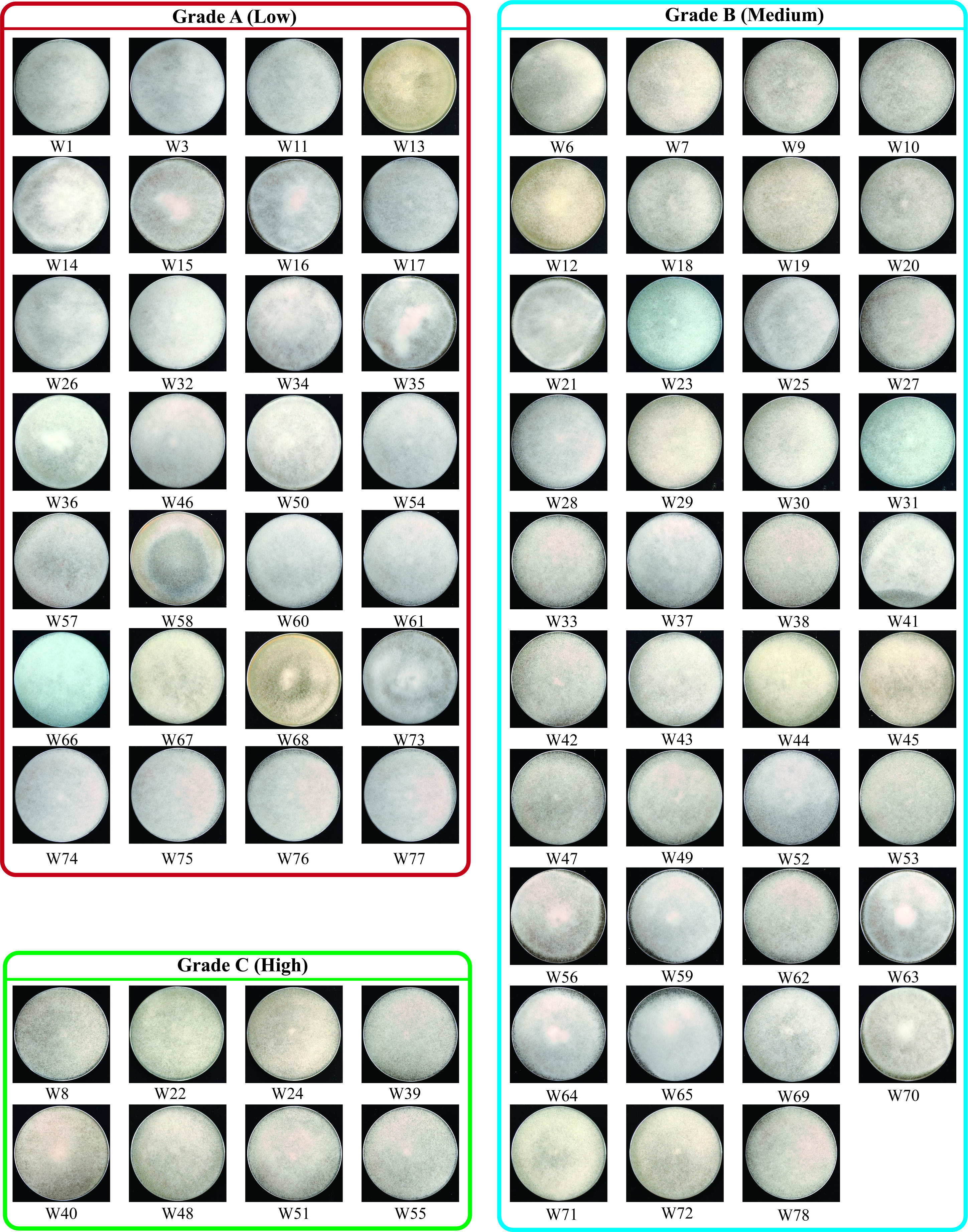

Supplement: Supplementary file 1 [file foods-14-00312-s001.zip › Figure S1 (Comprehensive classification of Rhizopus arrhizus strains based on sporulation levels).tif]
